# Rate differences between first and second primary cancers may outline immune dysfunction as a key risk factor

**DOI:** 10.1002/cam4.3454

**Published:** 2020-09-22

**Authors:** Guoqiao Zheng, Kristina Sundquist, Jan Sundquist, Asta Försti, Akseli Hemminki, Kari Hemminki

**Affiliations:** ^1^ Division of Molecular Genetic Epidemiology German Cancer Research Center (DKFZ) Heidelberg Germany; ^2^ Center for Primary Health Care Research Lund University Malmö Sweden; ^3^ Department of Family Medicine and Community Health Department of Population Health Science and Policy Icahn School of Medicine at Mount Sinai New York NY USA; ^4^ Center for Community‐based Healthcare Research and Education (CoHRE) Department of Functional Pathology School of Medicine Shimane University Shimane Japan; ^5^ Hopp Children's Cancer Center (KiTZ) Heidelberg Germany; ^6^ Division of Pediatric Neurooncology German Cancer Research Center (DKFZ) German Cancer Consortium (DKTK) Heidelberg Germany; ^7^ Cancer Gene Therapy Group Translational Immunology Research Program University of Helsinki Helsinki Finland; ^8^ Comprehensive Cancer Center Helsinki University Hospital Helsinki Finland; ^9^ Division of Cancer Epidemiology German Cancer Research Center (DKFZ) Heidelberg Germany; ^10^ Faculty of Medicine and Biomedical Center in Pilsen Charles University in Prague Pilsen Czech Republic

**Keywords:** cancer risk, immune suppression, multiple cancers, risk factors

## Abstract

**Background:**

Many cancers are increased in immunosuppressed patients and evidence is accumulating that immune dysfunction may be a contributing risk factor for second primary cancers (SPCs). The aim of this study was to explore the potential influence of immune mechanisms in SPC.

**Methods:**

We used the Swedish Cancer Registry (1990‐2015) to select 13 male and 14 female first primary cancers (FPCs) that are known to be related to immune suppression. We assessed relative risks (RRs) for any of these as concordant (same first and second cancer) and discordant FPC‐SPC pairs. Hierarchical clustering of significant RRs was performed for cancers as FPC and SPC.

**Results:**

Concordant risks for SPCs were excessive in men and women for nasal (RRs 59.3 for men and 150.6 for women), tongue/mouth (51.7 and 100.8), and lip (32.4 and 61.2) cancers. Heatmaps showed that some cancers, such as skin cancer, tongue/mouth cancers, and non‐Hodgkin lymphoma had multiple bidirectional associations as FPC and SPC. Nasal cancer and chronic lymphocytic leukemia had associations mainly as FPC while liver and kidney cancers showed most associations as SPC.

**Conclusions:**

Immune dysfunction may be a plausible contributing factor for most of the associations, which calls for experimental verification.

## Graphical abstract

Relative risks for a single second primary cancer after any first primary cancer (top), and RRs for any second primary cancer after a single first primary cancer (bottom). Male data are blue and female data in yellow symbols. The vertical bars show 95%CIs. NHL, non‐Hodgkin lymphoma, CLL, chronic lymphocytic leukemia.

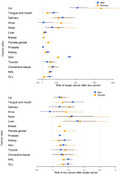

## 1 INTRODUCTION

A report of multiple primary cancers in cancer patients dating back to 1921 stated that 4.7% of cancers appeared to be “of multiple growth”.^1^ Since then improvements in cancer survival have increased the probability of second primary cancers (SPCs) and higher order primaries. However, for most first primary cancers (FPCs) higher order primaries are rare, and thus, the focus has been on SPCs.^2^, ^3^ The interest has been mainly twofold: one is a clinical interest because potentially carcinogenic therapies need to be balanced in favor of survival benefit, particularly in cancers of high cure rates, including Hodgkin lymphoma and testicular cancer.^4^, ^5^ The second interest is in understanding human carcinogenesis because, in addition to atomic bomb survivors, smokers, and some occupational settings, radio‐ and chemotherapy offer quantitative data on cancer risks in humans, including effects of dose, duration of exposure, and time from exposure to diagnosis.^6^, ^7^, ^8^ Outside a therapeutic setting, it is assumed that the causes of SPCs are the same as those of FPCs, including smoking, infections, and family history.^8^ Thus, understanding causes of SPC may advance understanding of causes of cancer in general. Recent molecular and clinical data provide evidence of the cross talk between the immune system and cancer cells, which involves, for example, local antitumor immune cell recruitment, tumor immunosuppression, immunoevasion, and the effects of chronic inflammation in carcinogenesis.^9^, ^10^ It is well‐known that iatrogenic immune suppression promotes appearance of many cancers, including squamous cell carcinoma (SCC) of the skin, nasal, and kidney cancers, non‐Hodgkin lymphoma (NHL), and cancers in the oral cavity.^11^, ^12^, ^13^, ^14^, ^15^ It is conceivable that FPC may negatively influence immune function by suppressing antitumor defense mechanisms in ways that may mimic iatrogenic immune suppression.^16^, ^17^, ^18^, ^19^ Such a mechanism would thus promote SPCs that are known to be in excess in immunosuppressed organ transplantation patients.^20^, ^21^, ^22^

The main hypothesis of the study is that immune mechanisms contribute to the appearance of SPCs. In order to provide the evidence on the potential influence of immune mechanisms in SPC, we systematically compared relative risks (RRs) for immune responsive cancers bidirectionally, that is, when they are FPCs and SPCs.^21^, ^22^ Cancers were identified from the Swedish Cancer Registry, and for immune responsive cancers, we selected those that were increased in a Swedish‐Danish study on organ transplant recipients ^14^; RRs for all cancers were increased 3.5 times in Sweden and 2.9 times in Denmark. As additional analyses, we assessed risks for “a single SPC after any FPC” and for “any SPC after a single FPC.” The former is a measure of collective carcinogenic factors (eg, immune dysfunction) FPCs exert on a single SPCs; the latter shows the influence of single factors (eg, carcinogenic therapy) on multiple SPCs.

## 2 PATIENTS AND METHODS

The Swedish Family‐Cancer Database, which was updated in 2015, includes data on more than 2.0 million cancer patients. Cancer diagnosis was recorded based on the 7th International Classification of Disease (ICD‐7) and its later versions. The data originate from the Swedish Cancer Register which was founded in 1958.^23^ It is compulsory for every health‐care provider to report newly detected cancer cases to a regional registry which are located at the oncological centers in each of six medical regions. The regional registries follow the same rules of registration, and carry out coding, checking, and correction of the records. All new incident tumors are registered as separate entities regardless of the morphology. Whether a tumor is a novel neoplasia, and thus, reportable, or a recurrence of an earlier diagnosed cancer is subject to the clinician's evaluation.^23^ This practice implies that concordant cancers in the same organ system are registers, deviating from the rules of the International Agency for Research on Cancer (IARC).^23^ However, these deviating rules only apply to the analyses shown in Table 1 while all other data are consistent with the IARC rules.

**TABLE 1:** Risk of second primary cancers on the concordant sites after the first year of first cancer diagnosis in men and women

Cancer site	Men					Women				
	N1	N2	RR	95% CI		N1	N2	RR	95% CI	
Lip	2010	36	32.4	23.2	45.2	1045	14	61.2	36.1	104.0
Tongue and mouth	4386	100	51.7	42.4	63.1	3398	127	100.8	84.3	120.4
Salivary	1051	0	0	0		1006	0	0	0	
Anus	803	3	37.5	12.0	116.5	1819	4	9.3	3.5	24.9
Nose	831	5	59.3	24.6	142.9	573	6	150.6	67.3	337.1
Liver	10 990	20	4.3	2.8	6.7	10 939	12	2.8	1.6	4.8
Breast		—	—	—	—	143 819	5759	1.8	1.8	1.9
Female genital		—	—	—	—	4450	47	17.4	13.1	23.3
Prostate	185 081	106	0.01	0.01	0.01	1045	—	—	—	—
Kidney	13 922	68	2.9	2.3	3.7	9520	64	5.8	4.6	7.5
SCC of the skin	28 324	3626	13.2	12.7	13.6	23 032	2136	13.7	13.1	14.3
Thyroid	2213	4	8.4	3.2	22.5	5758	8	2.5	1.2	5.0
Connective tissue	3564	11	8.5	4.7	15.4	2903	14	19.7	11.6	33.3
NHL	18 414	78	2.00	1.6	2.5	15 152	55	2.0	1. 6	2.6
CLL	5828	3	0.5	0.2	1.5	3844	5	1.9	0. 8	4.5

Abbreviations: CLL, chronic lymphocytic leukemia; N1, number of first primary cancer; N2, number of second primary cancer; NHL, non‐Hodgkin lymphoma; SCC, squamous cell carcinoma.

The definition of “immune responsive cancer” was adopted from the Swedish‐Danish study on 20 804 solid organ transplant recipients.^14^ Immune responsive cancers were those with RRs over the mean RR of 3.2 for the two countries combined. In addition, chronic lymphocytic leukemia (CLL) was included because it was not included in the above study, but CLL is known to be immune responsive.^24^ Thus, cancers were included as immunosuppressive cancers: lip, tongue + mouth, salivary glands, anus, nose, liver, other female genitals (including vagina and vulva), kidney, skin (only squamous cell carcinoma, SCC), thyroid gland, connective tissue, and hematological tissues (NHL and CLL). Breast and prostate were included as reference sites as these are not considered immune responsive; in the above Swedish‐Danish study the RRs for breast cancer were 1.1 (0.9‐1.4) in Sweden and 1.3 (1.1‐1.7) in Denmark, for prostate cancer they were 0.9 (0.8‐1.1) and 1.0 (0.7‐1.4), respectively. Follow‐up was from 1990 through 2015, and was ended at diagnosis of SPC, immigration or death, whichever came earliest.

RRs for SPC were estimated through the incidence of second immune responsive cancer divided by the incidence of same cancer diagnosed as FPC in the general population. When risks for concordant cancers were considered, the diagnoses in the first year were deleted while for discordant sites all diagnoses were considered. The estimation was done separately for men and women and adjusted for age, calendar year, place of residence (four groups: big cities, South Sweden, North Sweden, or unspecified), and socioeconomic factors (six groups: blue‐collar worker, white‐collar worker, farmer, private, professional, or other/unspecified). Poisson regression model was employed for the risk estimation. For a collective assessment of the entire set of cancers, we calculated risks for a “single SPC after any FPC” (RR calculated for the single SPC) and, conversely, for “any SPC after a single FPC” (RR calculated all SPC, excluding concordant, breast, and prostate cancers) separately for men and women. In these analyses, concordant cancers as well as breast and prostate cancers were not included as these would have skewed the results: concordant cancers with high risks and breast and prostate cancers with low risk and vast case numbers.

Hierarchical clustering of significant RRs was performed for cancers as FPC and SPC. The cluster was based on “Euclidean” by using R package “pheatmap.” The statistical tests were two‐tailed and *P* value < .05 was regarded as significant. All the analyses were performed in SAS 9.4 and figures were generated in R 3.5.

The study was approved by the local ethical committee without requirement for informed consent.

## 3 RESULTS

The data on concordant cancers are shown in Table 1, giving the numbers of FPCs and of SPCs, RRs and 95% confidence intervals (95%CIs). For 13 male cancers, case numbers ranged from 803 (anus) to 28 324 (skin SCC); 185 081 prostate cancers constituted the reference site. For 14 female cancers, nasal cancer was identified in 573 patients and that of skin cancer in 23 032 patients; the reference site of breast cancer had 143 819 patients. Among male cancers, RRs were increased for all but prostate cancer and CLL. Among female cancers, no increase was observed for CLL; salivary gland cancers had no SPCs. Very high risks of 10 or more were observed for lip (RR of 32.4 for men and 61.2 for women), tongue/mouth (51.7, 100.8), anal (37.5 and 9.3), nasal (59.3 and 150.6), female genital (17.4), skin SCC (13.2, 13.7), and connective tissue (8.5, 19.7) cancers. The RRs were identical between sexes for skin SCC and also for NHL but they were significantly different (95%CIs did not overlap) for tongue/mouth and kidney cancers, female RRs being twofold higher than the male RRs. Large but nonsignificant sex differences were observed for anal and thyroid cancers (male excess), and for lip, nasal, and connective tissue cancers (female excess). These results were based on follow‐up that commenced 1 year after FPC; when follow‐up was started immediately after the diagnosis of FPC, the RRs were much higher for salivary gland, kidney, and thyroid cancers while for other SPC the RRs were not different, considering sample sizes (data not shown).

Results for discordant male and female analyses are shown in Table S1. Results are shown when any sex‐specific RR was significant.

**FIGURE 1:**
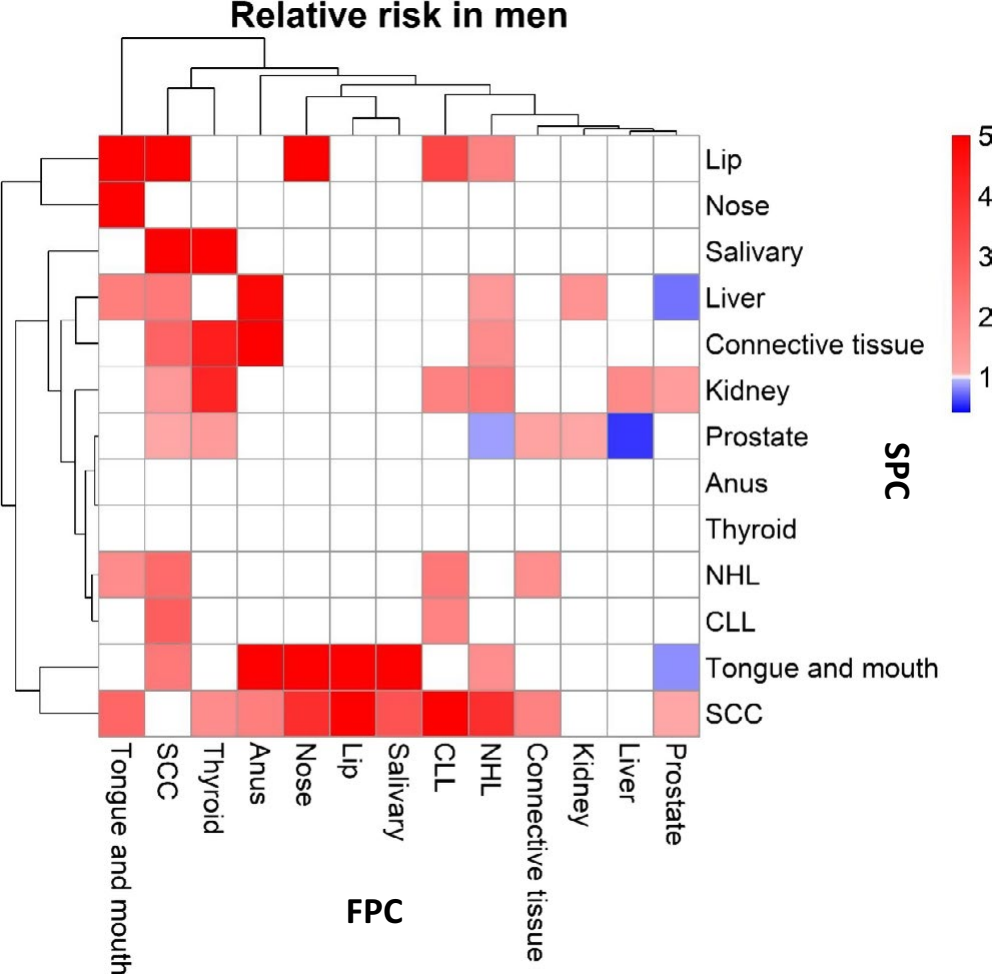
Heatmap of RRs for male first primary cancers (FPC, vertical, cancer listed in the bottom) and second primary cancers (SPC, horizontal, cancer listed on the right). The RR scale is shown in the top right corner. Only significant associations (95%CIs not overlapping with 1.00) were included; RRs of the insignificant or concordant associations were assigned as 1. NHL, non‐Hodgkin lymphoma, CLL, Chronic lymphocytic leukemia

Based on the significant male RRs, a heatmap based on hierarchical clustering is presented as Figure 1 (note that concordant associations were left blank). The figure clearly shows that FPCs (read in vertical direction) of the tongue/mouth, skin, thyroid, and anus had many strong associations and NHL had also many but weaker associations. Skin, and tongue/mouth cancers were associated with most other sites as SPCs (horizontal direction); kidney cancer was also a common SPC but the associations were relatively weak. Based on FPCs, clustering was observed on the top of the heatmap, including cancers of the tongue/mouth, skin, thyroid, and anus. Similarly, cancers of the nose, lip, and salivary glands clustered together, as well as CLL, NHL, and connective tissue cancer. Considering clustering by SPC (left side of the heatmap), lip and nasal cancer, NHL and CLL, and tongue/mouth cancer and skin clustered together. The reference site, prostate, showed weak associations with kidney as FPC as well as SPC.

**FIGURE 2:**
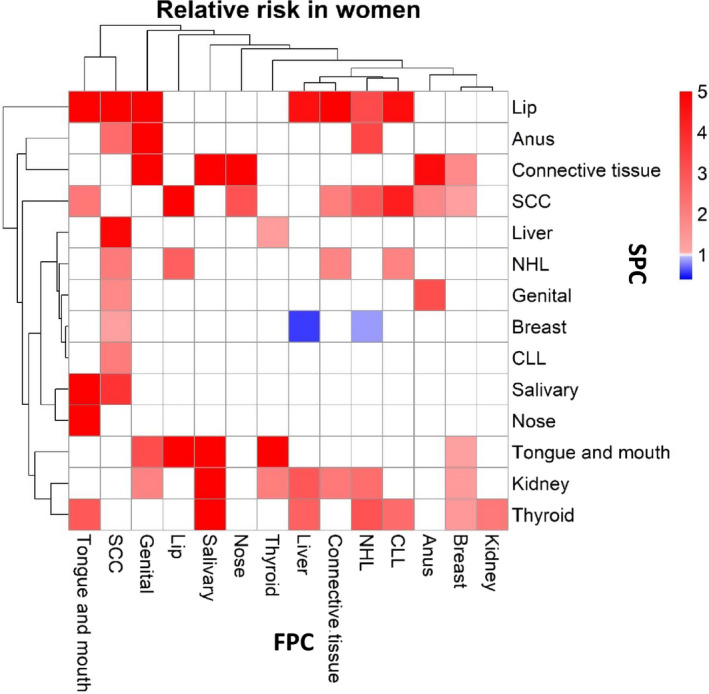
Heatmap of RRs for female first primary cancers (FPC, vertical, cancer listed in the bottom) and second primary cancers (SPC, horizontal, cancer listed on the right). The RR scale is shown in the top right corner. Only significant associations (95%CIs not overlapping with 1.00) were included; RRs of the insignificant or concordant associations were assigned as 1. NHL, non‐Hodgkin lymphoma, CLL, Chronic lymphocytic leukemia

In Figure 2, discordant female cancers are shown, and in agreement with male cancers, skin, tongue/mouth cancers, and NHL as FPCs show multiple associations. Female genital and salivary gland cancers show a few but strong associations. Lip, connective tissue, skin, tongue/mouth, and kidney cancer were common SPCs. Hierarchical clustering shows some similarities with male clustering. FPCs of the tongue/mouth and skin cancers clustered together (top of the heatmap), and were joined by female genital, lip, and salivary gland cancers. Liver and connective tissue cancers clustered, as did NHL and CLL. SPC clusters (left side of the heatmap) include cancer of the salivary glands and nose as one, and lip, anal, connective tissue, and skin cancers as another cluster. The reference site breast showed weak associations as FPC as well as SPC.

**FIGURE 3:**
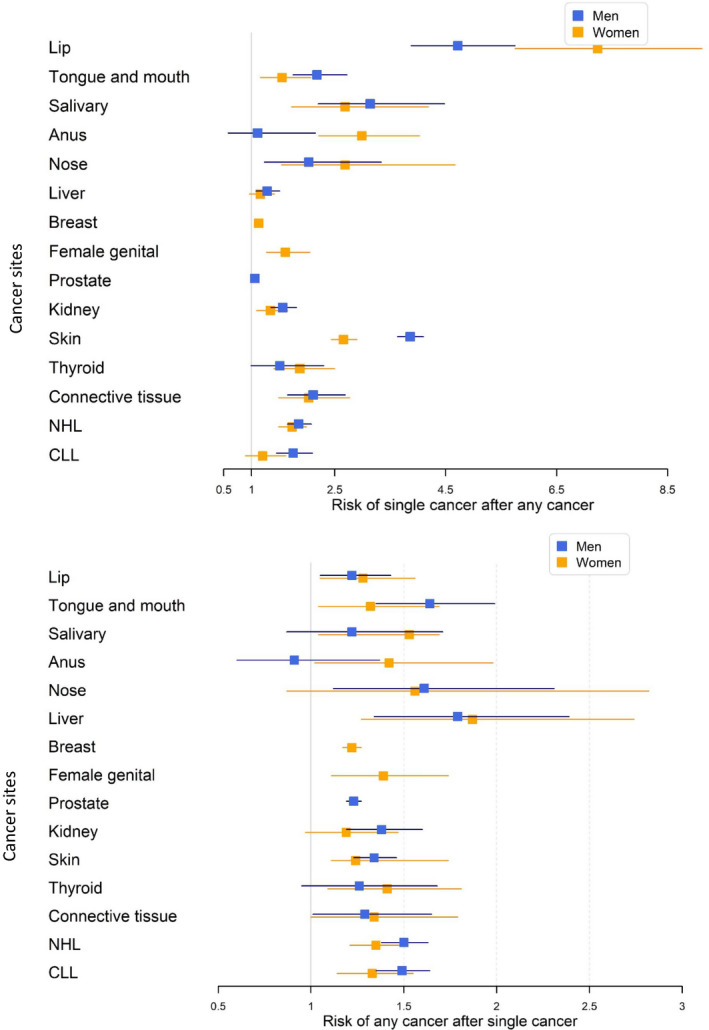
RRs for a single second primary cancer after any first primary cancer (top), and RRs for any second primary cancer after a single first primary cancer (bottom). Male data are blue and female data in yellow symbols. The vertical bars show 95%CIs. NHL, non‐Hodgkin lymphoma, CLL, Chronic lymphocytic leukemia

We assessed risks for “a single SPC after any FPC” and, conversely, for “any SPC after a single FPC” for men and women (Figure 3). The detailed data are presented as Tables S2 and S3, respectively. Among “single SPC after any FPC,” the highest risks were noted for lip (7.24 female, 4.72 male), salivary gland, and skin cancers, the latter with a large male excess (3.86 vs 2.66). Anal cancer was only increased among women while liver cancer was increased only among men.

For “risk for any cancer after single cancer” (Figure 3), liver cancer (1.79 male, 1.87 female) ranked first, followed by nasal (only male RR of 1.61 was significant) and tongue/mouth cancers (1.64 and 1.32). For salivary gland and anal cancer only female RRs were significant.

## 4 DISCUSSION

The long‐suspected role of immune surveillance in human cancer has gained strong experimental support in the past decade and, even more gratifying, that these scientific breakthroughs have also benefited clinical oncology in the context of diagnostics, prognostication, drug development, and therapy.^10^, ^25^, ^26^, ^27^ The recent scientific discoveries on the role of immunological factors in cancer were accomplished a few decades later than it had become known that iatrogenic immune suppression poses a large risk of cancer.^11^, ^28^, ^29^ Why some cancers are particularly responsive to immune suppression or immunotherapy is not well understood but one general correlation with immune responsive tumors is their mutational load, although there are also others, for example, PDL1 expression and CD8+ cell infiltration.^30^ Further insight is being gained by detailed cell type classifications considering immune cell repertoire in tumor infiltration and stroma.^29^, ^30^, ^31^, ^32^ Immunoscores have been devised for clinical prognostication; for example, a successful immunoscore for colon cancer included counts of CD3+ and CD8+ T‐cells in the tumor and in in the invasive margin.^33^

The generally higher risk for SPC than for FPC, and the selective influence on particular cancers has been suggested to have immunological mechanisms.^8^, ^21^, ^22^ In the present study, we focused on cancers that showed a higher than average risk in immunosuppressed patients and provide strong epidemiological evidence on the role of immune factors in SPC causation. Many of the analyzed immune responsive cancers were rare and hardly any literature exists on these cancers in the context of SPCs, which is particularly true of the included solid cancers.

First, we demonstrated that concordant risks for SPCs were excessive both in men and women for nasal (RRs 59.3 for men and 150.6 for women), tongue/mouth (51.7 and 100.8), and lip (32.4 and 61.2) cancers and a few others. The access to concordant second cancers is rather unique to the Swedish Cancer Registry as SPCs are recorded even in the same organ system, opposite to the rule of IARC, as discussed under Methods. The caveat in concordant cancers is that one cannot exclude the possibility that SPC may be metastasis from FPC. However starting the follow‐up 1 year after diagnosis did not change the risk estimates for most of the cancers analyzed in this study; the exceptions were salivary gland, kidney, and thyroid cancers for which the immediate follow‐up showed higher risks than the subsequent one. It is known that for kidney cancer the bilateral tumor is often diagnosed synchronously with the first tumor.^34^ The causes for these cancers with a very high risk are not known and are probably interactions of immune dysfunction and external causes. Human papilloma viruses (HPVs) contribute to a minor portion of cancers in the oral cavity and large portions of female genital and anal cancers.^35^, ^36^ Epstein‐Barr virus is associated with nasopharyngeal cancer and may also contribute to cancer of the nasal cavity.^35^, ^36^ The very high risks should alert clinicians to a long‐term follow‐up of patients with these cancers; whether the diagnosed SPCs are actually metastases may not influence treatment.

The heatmaps showed that some cancers had multiple associations bidirectionally as FPC and SPC in men and women; these typically included skin SCC, tongue/mouth cancer, and NHL. Some cancers had associations mainly as FPC, including nasal cancer and CLL, and in male anal and thyroid cancers. A few cancers showed associations mainly as SPC, including liver and kidney cancers, and we can speculate that their immune functions are particularly influences by FPCs. Clustering analyses showed similarities between cancers either as FPC or SPC. FPC clusters included tongue/mouth and skin cancers, lip and salivary gland cancers, liver and connective tissue cancers, as well as NHL and CLL. SPC clusters include salivary glands and nose as one, and lip, anal, connective tissue, and skin cancers as another cluster.

The novel type of analysis “risk for a single SPC after any FPC” and “risk for any SPC after a single FPC” was revealing in many ways, although detection bias should not be excluded as a potential cause. First it showed that almost for any single cancer, the risks were significant indicating existence of common, collective carcinogenic factors. Notably, the highest increases were recorded for cancers of the lip, salivary gland, nose, and skin, known to respond after immune suppressive therapy. Among the reference cancers, prostate cancer was not increased after any cancer and the RR for breast cancer was only 1.13. Risk of “any SPC after single cancer” is likely to reflect treatment related effects and, accordingly, cancers of the skin, kidney, and oral cavity, for which primary treatment is surgery, were only modestly increased, at level no higher than breast and prostate cancers (1.22 and 1.23). Male and female risks did not differ for “any cancer after single cancer” but for “single cancer after any cancer” only female risk was significant for anal cancer and the male RR was higher than the female one for skin cancer.

The explanation for discordant associations is difficult to find among environmental risk factors for such a diverse set of cancers, including solid and hematological malignancies. Even if some of these malignancies are treated with chemo‐ and/or radiotherapy, many are at least initially treated with surgery (skin SCC, lip, tongue/mouth, salivary glands) and therapy‐related SPCs would not be very probable. Surveillance bias may generally influence SPCs, particularly in anatomic proximal to FPCs, but increased risks of diverse SPC such as depicted in Figure 3 suggest mainly other causes. Immune dysfunction may be a plausible contributing factor as the selection criteria for cancer types specified that had to be increased in immunosuppressed patients more than average cancers.

The present study was nation‐wide but many of the included cancer sites were rare with the consequence of limited statistical power. Another limitation was the number of comparisons whereby chance findings were unavoidable. As limited literature has been published SPC in a similar context, it was not possible to conform the finding through the existing literature. However, the applied bidirectional analysis helped to some extent to alert about chance findings. Finally, the definition of “immune responsive cancer” was operational and based on the findings from excess cancers in immunosuppressed organ transplant patients.

In conclusion, the results show novel associations of many rare cancers in the setting of SPCs that are potentially related to immunological response. Cancers of the lip, tongue/mouth, salivary glands, nose, connective tissue, and CLL were found to be associated with many SPCs with high risk and many of them presented also high risks as SPCs. The results call for experimental studies to search for the putative immunological mechanisms and mechanisms of carcinogenesis influencing SPC risks. The high risks recorded should justify vigilance SPC surveillance, and help target follow‐up to high‐risk SPCs.

## CONFLICTS OF INTEREST

AH is a shareholder in Targovax ASA. AH is employee and shareholder in TILT Biotherapeutics Ltd. Other authors declared no conflict of interest.

## AUTHOR CONTRIBUTIONS

Design: KH, GZ; Acquisition of data: JS, KS; Statistical analysis and interpretation: GZ, KH, AF, AH, JS, KS. Manuscript writing: KH and all other authors. Approval of the final text: All authors.

## Supporting information

Table S1‐3

## Data Availability

The data that support the findings of this study are available from Lund University but restrictions apply to the availability of these data, which were used under license for the current study and so are not publicly available.
