# Supplementary material for: Rate differences between first and second primary cancers may outline immune dysfunction as a key risk factor
Source: Cancer Med. 2020 Sep 22;9(21):8258–65. doi: 10.1002/cam4.3454 (PMC7643639; doi:10.1002/cam4.3454)
Supplement: Supplementary file 1 — Table S1‐3 [file CAM4-9-8258-s001.docx]

Supplementary Table 1 Risk of second primary cancers on the discordant sites in men and women

| First primary cancer sites | Second primary cancer sites | Men | | | | | Women | | | | |
| --- | --- | --- | --- | --- | --- | --- | --- | --- | --- | --- | --- |
|  |  | N1 | N2 | RR | 95% CI | | N1 | N2 | RR | 95% CI | |
| Lip | Tongue and mouth | 4386 | 12 | 7.02 | 3.98 | 12.39 | 3398 | 4 | 5.94 | 2.23 | 15.83 |
|  | SCC | 28324 | 148 | 5.96 | 5.07 | 7.01 | 23032 | 93 | 11.38 | 9.28 | 13.95 |
|  | NHL | 18414 | 13 | 1.49 | 0.86 | 2.57 | 15152 | 8 | 2.74 | 1.37 | 5.48 |
| Tongue  and  mouth | Lip | 2010 | 12 | 10.54 | 5.98 | 18.6 | 1045 | 6 | 12.17 | 5.45 | 27.17 |
|  | Salivary | 1051 | 1 | 1.89 | 0.27 | 13.41 | 1006 | 2 | 5.68 | 1.42 | 22.77 |
|  | Nose | 831 | 5 | 11.48 | 4.76 | 27.67 | 10939 | 2 | 8.86 | 2.21 | 35.55 |
|  | Liver | 10990 | 13 | 2.03 | 1.18 | 3.5 | 573 | 7 | 1.47 | 0.70 | 3.08 |
|  | SCC | 28324 | 51 | 2.59 | 1.97 | 3.41 | 23032 | 30 | 2.21 | 1.55 | 3.16 |
|  | Thyroid | 2213 | 2 | 2.29 | 0.57 | 9.18 | 5758 | 4 | 2.93 | 1.10 | 7.81 |
|  | NHL | 18414 | 17 | 1.72 | 1.07 | 2.77 | 15152 | 11 | 1.72 | 0.95 | 3.11 |
| Salivary | Tongue and mouth | 4386 | 5 | 7.14 | 2.97 | 17.17 | 3398 | 3 | 5.7 | 1.84 | 17.69 |
|  | Kidney | 13922 | 5 | 2.09 | 0.87 | 5.03 | 9520 | 8 | 5.95 | 2.97 | 11.9 |
|  | SCC | 28324 | 23 | 3.09 | 2.06 | 4.66 | 23032 | 7 | 1.44 | 0.68 | 3.01 |
|  | Thyroid | 2213 | 1 | 3.64 | 0.51 | 25.85 | 5758 | 6 | 10.5 | 4.72 | 23.4 |
|  | Connective tissue | 3564 | 2 | 3.7 | 0.92 | 14.8 | 2903 | 2 | 5.53 | 1.38 | 22.13 |
| Anus | Tongue and mouth | 4386 | 3 | 6.34 | 2.04 | 19.67 | 3398 | 2 | 2.14 | 0.54 | 8.57 |
|  | Liver | 10990 | 6 | 4.83 | 2.17 | 10.75 | 573 | 0 | 0 | 0 |  |
|  | Female genital | - | - | - | - | - | 4450 | 4 | 3.15 | 1.18 | 8.4 |
|  | SCC | 28324 | 10 | 2.06 | 1.11 | 3.82 | 23032 | 16 | 1.84 | 1.13 | 3.01 |
|  | Connective tissue | 3564 | 2 | 5.69 | 1.42 | 22.75 | 2903 | 3 | 4.75 | 1.53 | 14.74 |
| Liver | Lip | 2010 | 1 | 0.61 | 0.09 | 4.36 | 1045 | 3 | 4.64 | 1.49 | 14.42 |
|  | Breast | - | - | - | - | - | 143819 | 38 | 0.58 | 0.42 | 0.8 |
|  | Prostate | 185081 | 84 | 0.56 | 0.46 | 0.7 | - | - | - | - | - |
|  | Kidney | 13922 | 19 | 1.77 | 1.13 | 2.78 | 9520 | 20 | 2.96 | 1.91 | 4.6 |
|  | Thyroid | 2213 | 3 | 2.79 | 0.9 | 8.66 | 5758 | 5 | 2.71 | 1.13 | 6.51 |
| Nose | Lip | 2010 | 2 | 6.98 | 1.74 | 27.92 | 1045 | 0 | 0 | 0 |  |
|  | Tongue and mouth | 4386 | 3 | 6.03 | 1.94 | 18.7 | 3398 | 0 | 0 | 0 |  |
|  | SCC | 28324 | 20 | 3.89 | 2.51 | 6.04 | 23032 | 8 | 3.09 | 1.55 | 6.19 |
|  | Connective tissue | 3564 | 1 | 2.66 | 0.37 | 18.86 | 2903 | 2 | 10.36 | 2.59 | 41.44 |
| Breast | Tongue and mouth | - | - | - | - | - | 3398 | 111 | 1.26 | 1.05 | 1.53 |
|  | Kidney | - | - | - | - | - | 9520 | 318 | 1.35 | 1.2 | 1.51 |
|  | SCC | - | - | - | - | - | 23032 | 954 | 1.27 | 1.19 | 1.35 |
|  | Thyroid | - | - | - | - | - | 5758 | 128 | 1.45 | 1.22 | 1.73 |
|  | Connective tissue | - | - | - | - | - | 2903 | 105 | 1.76 | 1.44 | 2.14 |
| Female genitals | Lip | - | - | - | - | - | 1045 | 4 | 5.28 | 1.98 | 14.09 |
|  | Tongue and mouth | - | - | - | - | - | 3398 | 7 | 3.21 | 1.53 | 6.73 |
|  | Anus | - | - | - | - | - | 1819 | 13 | 12.43 | 7.2 | 21.47 |
|  | Kidney | - | - | - | - | - | 9520 | 11 | 1.93 | 1.07 | 3.49 |
|  | Connective tissue | - | - | - | - | - | 2903 | 8 | 5.39 | 2.69 | 10.8 |
| Prostate | Tongue and mouth | 4386 | 132 | 0.82 | 0.69 | 0.98 | - | - | - | - | - |
|  | Liver | 10990 | 376 | 0.74 | 0.67 | 0.83 | - | - | - | - | - |
|  | Kidney | 13922 | 768 | 1.31 | 1.22 | 1.41 | - | - | - | - | - |
|  | SCC | 28324 | 2339 | 1.1 | 1.05 | 1.15 | - | - | - | - | - |
| Kidney | Liver | 10990 | 39 | 1.58 | 1.15 | 2.16 | 573 | 1 | 1.36 | 0.19 | 9.66 |
|  | Prostate | 185081 | 535 | 1.13 | 1.04 | 1.23 | - | - | - | - | - |
|  | Thyroid | 2213 | 6 | 1.95 | 0.87 | 4.34 | 5758 | 9 | 2.16 | 1.12 | 4.16 |
| Skin | Lip | 2010 | 83 | 7.48 | 5.98 | 9.35 | 1045 | 61 | 12.4 | 9.51 | 16.16 |
|  | Tongue and mouth | 4386 | 33 | 2.17 | 1.53 | 3.06 | 3398 | 14 | 1.11 | 0.65 | 1.87 |
|  | Salivary | 1051 | 27 | 6.01 | 4.07 | 8.88 | 1006 | 11 | 3.78 | 2.08 | 6.89 |
|  | Anus | 803 | 7 | 2.06 | 0.97 | 4.36 | 1819 | 14 | 2.47 | 1.46 | 4.2 |
|  | Nose | 831 | 7 | 2.16 | 1.02 | 4.56 | 10939 | 42 | 1.23 | 0.91 | 1.66 |
|  | Liver | 10990 | 55 | 1.19 | 0.91 | 1.55 | 573 | 9 | 4.88 | 2.51 | 9.5 |
|  | Breast | - | - | - | - | - | 143819 | 440 | 1.23 | 1.12 | 1.35 |
|  | Female genital | - | - | - | - | - | 4450 | 35 | 1.81 | 1.3 | 2.53 |
|  | Prostate | 185081 | 1205 | 1.11 | 1.05 | 1.18 | - | - | - | - | - |
|  | Kidney | 13922 | 74 | 1.44 | 1.14 | 1.81 | 9520 | 25 | 0.96 | 0.65 | 1.42 |
|  | Connective tissue | 3564 | 38 | 2.67 | 1.93 | 3.69 | 2903 | 13 | 1.66 | 0.96 | 2.86 |
|  | NHL | 18414 | 203 | 2.47 | 2.15 | 2.84 | 15152 | 111 | 2.13 | 1.77 | 2.57 |
|  | CLL | 5828 | 87 | 2.79 | 2.25 | 3.45 | 3844 | 32 | 2.1 | 1.48 | 2.97 |
| Thyroid | Tongue and mouth | 4386 | 1 | 0.8 | 0.11 | 5.65 | 3398 | 2 | 7.76 | 3.22 | 18.69 |
|  | Salivary | 1051 | 2 | 6.74 | 1.68 | 27 | 1006 | 5 | 0.77 | 0.11 | 5.45 |
|  | Liver | 10990 | 3 | 0.93 | 0.3 | 2.87 | 573 | 1 | 1.34 | 1.13 | 1.58 |
|  | Prostate | 185081 | 85 | 1.35 | 1.09 | 1.67 | - | - | - | - | - |
|  | Kidney | 13922 | 17 | 4.14 | 2.57 | 6.66 | 9520 | 13 | 2.06 | 1.2 | 3.55 |
|  | SCC | 28324 | 17 | 1.71 | 1.06 | 2.75 | 23032 | 17 | 1.02 | 0.64 | 1.64 |
|  | Connective tissue | 3564 | 4 | 4.32 | 1.62 | 11.53 | 2903 | 4 | 2.28 | 0.85 | 6.08 |
| Connective tissue | Lip | 2010 | 1 | 0.96 | 0.14 | 6.84 | 1045 | 2 | 5.01 | 1.25 | 20.04 |
|  | Prostate | 185081 | 131 | 1.2 | 1.01 | 1.42 | - | - | - | - | - |
|  | Kidney | 13922 | 9 | 1.38 | 0.72 | 2.64 | 9520 | 7 | 2.18 | 1.04 | 4.57 |
|  | SCC | 28324 | 40 | 1.97 | 1.44 | 2.68 | 23032 | 22 | 2.06 | 1.35 | 3.12 |
|  | NHL | 18414 | 15 | 1.68 | 1.01 | 2.79 | 15152 | 10 | 1.89 | 1.01 | 3.51 |
| NHL | Lip | 2010 | 11 | 1.96 | 1.08 | 3.54 | 1045 | 8 | 3.24 | 1.61 | 6.5 |
|  | Tongue and mouth | 4386 | 17 | 1.65 | 1.02 | 2.66 | 3398 | 9 | 1.25 | 0.65 | 2.41 |
|  | Anus | 803 | 3 | 1.47 | 0.47 | 4.57 | 1819 | 12 | 3.35 | 1.9 | 5.91 |
|  | Liver | 10990 | 46 | 1.46 | 1.1 | 1.96 | 573 | 1 | 0.88 | 0.12 | 6.25 |
|  | Breast | - | - | - | - | - | 143819 | 215 | 0.86 | 0.75 | 0.98 |
|  | Prostate | 185081 | 516 | 0.87 | 0.8 | 0.95 | - | - | - | - | - |
|  | Kidney | 13922 | 82 | 2.25 | 1.81 | 2.8 | 9520 | 49 | 2.44 | 1.85 | 3.24 |
|  | SCC | 28324 | 413 | 3.92 | 3.55 | 4.32 | 23032 | 196 | 2.95 | 2.56 | 3.4 |
|  | Thyroid | 2213 | 7 | 1.72 | 0.82 | 3.61 | 5758 | 21 | 3.08 | 2.01 | 4.73 |
|  | Connective tissue | 3564 | 14 | 1.74 | 1.03 | 2.94 | 2903 | 6 | 1.22 | 0.55 | 2.71 |
| CLL | Lip | 2010 | 8 | 3.38 | 1.69 | 6.77 | 1045 | 4 | 4.66 | 1.74 | 12.44 |
|  | Prostate | 185081 | 218 | 0.89 | 0.78 | 1.02 | - | - | - | - | - |
|  | Kidney | 13922 | 28 | 1.95 | 1.35 | 2.83 | 9520 | 11 | 1.71 | 0.95 | 3.09 |
|  | SCC | 28324 | 336 | 7.1 | 6.37 | 7.91 | 23032 | 106 | 4.32 | 3.57 | 5.22 |
|  | Thyroid | 2213 | 1 | 0.66 | 0.09 | 4.7 | 5758 | 5 | 2.5 | 1.04 | 6.02 |
|  | Connective tissue | 3564 | 7 | 2.2 | 1.05 | 4.63 | 2903 | 2 | 1.26 | 0.31 | 5.04 |
|  | NHL | 18414 | 37 | 1.91 | 1.38 | 2.63 | 15152 | 21 | 1.94 | 1.27 | 2.98 |

N1, number of first primary cancer patients, N2, number of second primary cancer patients, NHL, non-Hodgkin lymphoma, CLL, chronic lymphocytic leukemia

Supplementary Table 2 Risk of single immune responsive cancer after any cancers in men and women

|  | Men | | | | | Women | | | | |
| --- | --- | --- | --- | --- | --- | --- | --- | --- | --- | --- |
| Cancersite | N1 | N2 | RR | 95% CI | | N1 | N2 | RR | 95% CI | |
| Lip | 2010 | 107 | 4.72 | 3.88 | 5.75 | 1045 | 80 | 7.24 | 5.75 | 9.12 |
| Tongue and mouth | 4386 | 82 | 2.18 | 1.75 | 2.72 | 3398 | 47 | 1.55 | 1.16 | 2.07 |
| Salivary | 1051 | 32 | 3.14 | 2.2 | 4.48 | 1006 | 20 | 2.69 | 1.72 | 4.19 |
| Anus | 803 | 9 | 1.11 | 0.58 | 2.15 | 1819 | 44 | 2.99 | 2.21 | 4.03 |
| Liver | 10990 | 145 | 1.28 | 1.08 | 1.51 | 10939 | 109 | 1.16 | 0.96 | 1.41 |
| Nose | 831 | 16 | 2.03 | 1.23 | 3.34 | 573 | 13 | 2.69 | 1.54 | 4.67 |
| Breast | - | - | - | - | - | 143819 | 1173 | 1.13 | 1.07 | 1.2 |
| Female genital | - | - | - | - | - | 4450 | 69 | 1.61 | 1.27 | 2.05 |
| Prostate | 185081 | 2623 | 1.06 | 1.02 | 1.1 | - | - | - | - | - |
| Kidney | 13922 | 176 | 1.56 | 1.35 | 1.81 | 9520 | 91 | 1.34 | 1.09 | 1.65 |
| Skin | 28324 | 1098 | 3.86 | 3.63 | 4.1 | 23032 | 521 | 2.66 | 2.44 | 2.9 |
| Thyroid | 2213 | 22 | 1.51 | 0.99 | 2.3 | 5758 | 47 | 1.87 | 1.40 | 2.5 |
| Connective tissue | 3564 | 66 | 2.11 | 1.65 | 2.69 | 2903 | 41 | 2.03 | 1.49 | 2.77 |
| NHL | 18414 | 286 | 1.85 | 1.65 | 2.08 | 15152 | 191 | 1.73 | 1.49 | 1.99 |
| CLL | 5828 | 115 | 1.75 | 1.45 | 2.1 | 3844 | 44 | 1.2 | 0.89 | 1.62 |

Any cancers include all the immune responsive cancers, N1, number of first primary cancer, N2, number of second primary cancer, NHL, non-Hodgkin lymphoma, CLL, chronic lymphocytic leukemia

Supplementary Table 3 Risk of any cancers after single immune responsive cancer in men and women

|  | Men | | | | | Women | | | | |
| --- | --- | --- | --- | --- | --- | --- | --- | --- | --- | --- |
| Cancersite | N1 | N2 | RR | 95% CI | | N1 | N2 | RR | 95% CI | |
| Lip | 90326 | 163 | 1.22 | 1.05 | 1.43 | 82394 | 97 | 1.28 | 1.05 | 1.56 |
| Tongue and mouth | 87950 | 100 | 1.64 | 1.35 | 1.99 | 80041 | 65 | 1.32 | 1.04 | 1.69 |
| Salivary | 91285 | 33 | 1.22 | 0.87 | 1.71 | 82433 | 26 | 1.53 | 1.04 | 2.25 |
| Anus | 91533 | 23 | 0.91 | 0.6 | 1.37 | 81620 | 35 | 1.42 | 1.02 | 1.98 |
| Liver | 81346 | 46 | 1.79 | 1.34 | 2.39 | 72500 | 26 | 1.87 | 1.27 | 2.74 |
| Nose | 91505 | 29 | 1.61 | 1.12 | 2.31 | 82866 | 11 | 1.56 | 0.87 | 2.82 |
| Breast | - | - | - | - | - | 83439 | 2472 | 1.22 | 1.17 | 1.27 |
| Female genital | - | - | - | - | - | 78989 | 77 | 1.39 | 1.11 | 1.74 |
| Prostate | 92336 | 4589 | 1.23 | 1.19 | 1.27 | - | - | - | - | - |
| Kidney | 78414 | 182 | 1.38 | 1.19 | 1.6 | 73919 | 87 | 1.19 | 0.97 | 1.47 |
| Skin | 64012 | 517 | 1.34 | 1.23 | 1.46 | 60407 | 327 | 1.24 | 1.11 | 1.38 |
| Thyroid | 90123 | 48 | 1.26 | 0.95 | 1.68 | 77681 | 60 | 1.41 | 1.09 | 1.81 |
| Connective tissue | 88772 | 65 | 1.29 | 1.01 | 1.65 | 80536 | 45 | 1.34 | 1.00 | 1.79 |
| NHL | 73922 | 546 | 1.5 | 1.38 | 1.63 | 68287 | 300 | 1.35 | 1.21 | 1.51 |
| CLL | 86508 | 402 | 1.49 | 1.35 | 1.64 | 79595 | 161 | 1.33 | 1.14 | 1.55 |

Any cancers include all the immune responsive cancers, N1, number of first primary cancer, N2, number of second primary cancer, NHL, non-Hodgkin lymphoma, CLL, chronic lymphocytic leukemia
